# Extreme values of derivatives of zeta and *L*‐functions

**DOI:** 10.1112/blms.12915

**Published:** 2023-09-11

**Authors:** Daodao Yang

**Affiliations:** ^1^ Institute of Analysis and Number Theory Graz University of Technology Graz Austria

## Abstract

It is proved that as T→∞, uniformly for all positive integers $\ell ⩽\left(\log_{3}T\right)/\left(\log_{4}T\right)$, we have

$$\;\max_{T⩽t⩽2T}\left|\zeta^{\left(\ell \right)}\left(1+it\right)\right|⩾\left(Y_{\ell }+o\left(1\right)\right){\left(\log_{2}T\right)}^{\ell +1}\;,$$

where $Y_{\ell }=\int_{0}^{\infty }u^{\ell }\rho \left(u\right)du$. Here, ρ(u) is the Dickman function. We have $Y_{\ell }>e^{\gamma }/\left(\ell +1\right)$ and $\log\;Y_{\ell }=\left(1+o\left(1\right)\right)\ell \log\ell$ when ℓ→∞, which significantly improves previous results in [17, 40]. Similar results are established for Dirichlet *L*‐functions. On the other hand, when assuming the Riemann hypothesis and the generalized Riemann hypothesis, we establish upper bounds for $|\zeta^{\left(\ell \right)}\left(1+it\right)|$ and $|L^{\left(\ell \right)}\left(1,\chi \right)|$. Furthermore, when assuming the Granville–Soundararajan conjecture is true, we establish the following asymptotic formulas:

$$\;\max_{\begin{array}{c} \chi \neq \chi_{0}\\ \chi (\mathrm{mod}\;q) \end{array}}\left|L^{\left(\ell \right)}\left(1,\chi \right)\right|∼Y_{\ell }{\left(\log_{2}q\right)}^{\ell +1},\;\;\;\text{as}\;\;q\to \infty ,$$

where q is prime and ℓ∈N is given.

## 1 INTRODUCTION

This paper establishes the following results for extreme values of derivatives of the Riemann zeta function on the 1‐line. Throughout the paper, we define $Y_{\ell }=\int_{0}^{\infty }u^{\ell }\rho \left(u\right)du$ and ρ(u) denotes the Dickman function.

Theorem 1 As T→∞, uniformly for all positive integers $\ell ⩽\left(\log_{3}T\right)/\left(\log_{4}T\right)$, we have

$$\max_{T⩽t⩽2T}\left|\zeta^{\left(\ell \right)}\left(1+it\right)\right|⩾\left(Y_{\ell }+o\left(1\right)\right){\left(\log_{2}T\right)}^{\ell +1}\;.$$

Remark 1 By taking derivatives for the Laplace transform (see Lemma 2) of the Dickman function and applying Faà di Bruno's formula (e.g., see [11, page 134–137]), we can obtain a formula for $Y_{\ell }$ in terms of Bell polynomials. Namely, let $B_{\ell }\left(x_{1},x_{2},\text{…},x_{\ell }\right)$ be the ℓth complete exponential Bell polynomial, then we have $Y_{\ell }=e^{\gamma }{\left(-1\right)}^{\ell }B_{\ell }\left(-1,\frac{1}{2},\text{…},\frac{{\left(-1\right)}^{\ell }}{\ell }\right)$ . For instance, from $B_{1}\left(x_{1}\right)=x_{1}$, $B_{2}\left(x_{1},x_{2}\right)=x_{1}^{2}+x_{2}$, $B_{3}\left(x_{1},x_{2},x_{3}\right)=x_{1}^{3}+3x_{1}x_{2}+x_{3}$, we can compute  $Y_{1}=e^{\gamma }$, $Y_{2}=3e^{\gamma }/2$, and $Y_{3}=17e^{\gamma }/6$.

Remark 2 By the asymptotic formula (8), we have  $\log\;Y_{\ell }=\left(1+o\left(1\right)\right)\ell \log\ell$, as ℓ→∞.

Our result on the Riemann zeta function can be generalized to *L*‐functions. In the following theorem, we consider the case of Dirichlet *L*‐function L(s,χ) associated with nonprincipal characters χ(mod q).

Theorem 2 Let q be prime, then uniformly for all positive integers $\ell ⩽\log_{3}q/\log_{4}q$, we have

(1) $$\begin{array}{c} \max_{\begin{array}{c} \chi \neq \chi_{0}\\ \chi (\mathrm{mod}\;q) \end{array}}\left|L^{\left(\ell \right)}\left(1,\chi \right)\right|⩾\left(Y_{\ell }+o\left(1\right)\right){\left(\log_{2}q\right)}^{\ell +1},\;\;\;\text{as}\;\;q\to \infty . \end{array}$$

Remark 3 (1) The above result does not hold for general moduli q. For instance, assume $q=(\prod_{p⩽X}p)\cdot m$ , with m∈N and $X=\frac{1}{2}\log q$. This assumption will force χ(k)=0 if ∃p⩽X,p|k and thus will make $|L^{\left(\ell \right)}\left(1,\chi \right)|$ small. (2) However, if q is not divisible by small primes (e.g., consider the case that any prime factor of q is larger than $q^{\frac{1}{10}}$), then the above theorem will still hold. For simplicity, we state the result for prime moduli.

For upper bounds of $|\zeta^{\left(\ell \right)}\left(1+it\right)|$ and $|L^{\left(\ell \right)}\left(1,\chi \right)|$, we have following two results when assuming the Riemann hypothesis (RH) and the generalized Riemann hypothesis (GRH).

Theorem 3 Fix ℓ∈N. Assuming RH, we have

$$\begin{array}{c} \left|\zeta^{\left(\ell \right)}\left(1+it\right)\right|⩽\left(2^{\ell +1}\;Y_{\ell }+o\left(1\right)\right){\left(\log_{2}t\right)}^{\ell +1}\;,\;\text{as}\;t\to \infty \;. \end{array}$$

Theorem 4 Fix ℓ∈N. Let χ be any nonprincipal character (mod q), and assume GRH for L(s,χ). Then

$$\begin{array}{c} \left|L^{\left(\ell \right)}\left(1,\chi \right)\right|⩽\left(2^{\ell +1}\;Y_{\ell }+o\left(1\right)\right){\left(\log_{2}q\right)}^{\ell +1}\;,\;\text{as}\;q\to \infty \;. \end{array}$$

The key ingredient to prove Theorem 4 is the following theorem of Granville and Soundararajan [18, Theorem 2].

To state their result, we need some definitions. Let f be an arithmetic function. Define the functions Ψ(x,y) and Ψ(x,y;f) as

$$\begin{array}{c} \Psi \left(x,y\right):\;=\sum_{\begin{array}{c} n⩽x\\ P^{+}\left(n\right)⩽y \end{array}}1\;,\;\;\;\Psi \left(x,y;f\right):\;=\sum_{\begin{array}{c} n⩽x\\ P^{+}\left(n\right)⩽y \end{array}}f\left(n\right)\;. \end{array}$$

(Granville–Soundararajan) Let χ be any nonprincipal character (mod q), and assume the Riemann hypothesis for L(s,χ). If 1⩽x⩽q and $y⩾\log^{2}q\log^{2}x{\left(\log_{2}q\right)}^{12}$, then

$$\sum_{n⩽x}\chi \left(n\right)=\Psi \left(x,y;\chi \right)+O\left(\frac{\Psi (x,y)}{{\left(\log_{2}q\right)}^{2}}\right).$$

Further,

$$\left|\sum_{n⩽x}\chi \left(n\right)\right|\ll \Psi \left(x,\log^{2}q{\left(\log_{2}q\right)}^{20}\right),$$

and so, the following estimate holds:

$$\begin{array}{c} \left|\sum_{n⩽x}\chi \left(n\right)\right|=o\left(x\right)\;, \end{array}$$

when $\log x/\log_{2}q\to \infty$ as q→∞.

When $g\left(n\right)=n^{-it},\;\forall n\in N$, we write Ψ(x,y;t) in place of Ψ(x,y;g). Then, we have the following result analogous to the Granville–Soundararajan theorem.

Theorem 5 Assume RH and let T be sufficiently large. If 2⩽x⩽T, $T+y+3⩽t⩽T^{1000}$ and $y⩾\log^{2}T\log^{2}x{\left(\log_{2}T\right)}^{12}$, then

(2) $$\begin{array}{c} \sum_{n⩽x}\frac{1}{n^{it}}=\Psi \left(x,y;t\right)+O\left(\frac{\Psi (x,y)}{{\left(\log_{2}T\right)}^{2}}\right)\;. \end{array}$$

Further,

(3) $$\begin{array}{c} \left|\sum_{n⩽x}\frac{1}{n^{it}}\right|\ll \Psi \left(x,\log^{2}T{\left(\log_{2}T\right)}^{20}\right),\;\forall x\in \left[2,\;T\right]\;,\forall t\in \left[T+\log^{2}T{\left(\log_{2}T\right)}^{15},\;T^{1000}\right]\;, \end{array}$$

and so, the following estimate holds:

$$\begin{array}{c} \left|\sum_{n⩽x}\frac{1}{n^{it}}\right|=o\left(x\right)\;,\;\forall x\in \left[2,\;T\right]\;,\forall t\in \left[T+\log^{2}T{\left(\log_{2}T\right)}^{15},\;T^{1000}\right]\;, \end{array}$$

when $\log x/\log_{2}T\to \infty$ as T→∞.

In [18], Granville and Soundararajan also made the following conjecture.

(Granville–Soundararajan) There exists a constant A>0 such that for any nonprincipal character χ (mod q), and for any 1⩽x⩽q, we have, uniformly,

$$\sum_{n⩽x}\chi \left(n\right)=\Psi \left(x,y;\chi \right)+o\left(\Psi \left(x,y;\chi_{0}\right)\right),$$

where $y=\left(\log q+\log^{2}x\right){\left(\log_{2}q\right)}^{A}$.

A consequence of the Granville–Soundararajan conjecture is that the constant appearing in Theorem 2 is sharp.

Theorem 6 Assume the Granville–Soundararajan conjecture is true. Fix ℓ∈N. Let χ be any nonprincipal character (mod q), then

$$\begin{array}{c} \left|L^{\left(\ell \right)}\left(1,\chi \right)\right|⩽\left(Y_{\ell }+o\left(1\right)\right){\left(\log_{2}q\right)}^{\ell +1}\;,\;\text{as}\;q\to \infty \;. \end{array}$$

Combining with the lower bound, we immediately get the following asymptotic formulas.

Corollary 1 Assume the Granville–Soundararajan conjecture is true. Fix ℓ∈N. Let q be prime, then

$$\begin{array}{c} \max_{\begin{array}{c} \chi \neq \chi_{0}\\ \chi (\mathrm{mod}\;q) \end{array}}\left|L^{\left(\ell \right)}\left(1,\chi \right)\right|∼Y_{\ell }{\left(\log_{2}q\right)}^{\ell +1},\;\;\;\text{as}\;\;q\to \infty . \end{array}$$

We have the following analogous conjecture, which can imply that the constant appearing in Theorem 1 is sharp.

Conjecture 1 There exists a constant A>0 such that for any 1⩽x⩽T, 2T⩽t⩽5T, we have, uniformly,

$$\sum_{n⩽x}\frac{1}{n^{it}}=\Psi \left(x,y;t\right)+o\left(\Psi (x,y)\right)\;,\;\text{as}\;T\to \infty \;,$$

where $y=\left(\log T+\log^{2}x\right){\left(\log_{2}T\right)}^{A}$.

Theorem 7 Assume Conjecture 1 is true. Fix ℓ∈N. Then

$$\max_{T⩽t⩽2T}\left|\zeta^{\left(\ell \right)}\left(1+it\right)\right|∼Y_{\ell }{\left(\log_{2}T\right)}^{\ell +1},\;\text{as}\;T\to \infty \;.$$

The problem of obtaining extreme values of |ζ(1+it)| was first considered by Bohr and Landau, who established the result $\zeta \left(1+it\right)=\Omega \left(\log_{2}t\right)$ (see [38, Thm 8.5]) in 1910. In 1924, Littlewood (see [38, Thm 8.9(A)]) improved the result of Bohr and Landau, by proving that ${lim sup}_{t\to \infty }\left|\zeta \left(1+it\right)\right|/\left(\log_{2}t\right)⩾e^{\gamma }$. Littlewood's result has been improved in the past century by Levinson [30], by Granville–Soundararajan [21], and by Aistleitner–Mahatab–Munsch[2], who established that $\max_{\sqrt{T}⩽t⩽T}\left|\zeta \left(1+it\right)\right|⩾e^{\gamma }\left(\log_{2}T+\log_{3}T+C\right)$, for some constant C that can be effectively computed. Littlewood also established conditional results for the upper bound of |ζ(1+it)|. When assuming the truth of the RH, he proved that $\left|\zeta \left(1+it\right)\right|⩽\left(2e^{\gamma }+o\left(1\right)\right)\log_{2}t\;$, as t→∞ (see [38, Thm 14.9]). Furthermore, he conjectured that the maximum of |ζ(1+it)| on the interval [1,T] should satisfy the asymptotic formula $\max_{1⩽t⩽T}\left|\zeta \left(1+it\right)\right|∼e^{\gamma }\log_{2}T$. In [21], Granville and Soundararajan made the stronger conjecture that  $\max_{T⩽t⩽2T}\left|\zeta \left(1+it\right)\right|=e^{\gamma }\left(\log_{2}T+\log_{3}T+C\right)+o\left(1\right)$, for some constant C, which is also effectively computable.

When σ∈[1/2,1), the problem of obtaining extreme values of |ζ(σ+it)| also has a long history. For more background and results, see the recent survey [37] and [1, 4, 5, 6, 7, 8, 9, 14, 16, 21, 24, 32, 36, 39]. Here, we mention the recent breakthrough result by Bondarenko and Seip [5, 6] who prove that:

$$\max_{1⩽t⩽T}\left|\zeta \left(\frac{1}{2}+it\right)\right|⩾\exp\left(c\sqrt{\frac{\log T\log_{3}T}{\log_{2}T}}\right),\;\forall T\gg 1\;,$$

for any constant c<1. After refining methods of Bondarenko‐Seip, de la Bretèche, and Tenenbaum [14] show that any $c<\sqrt{2}$ is permissible in the above result.

In the past 5 years, the problem of obtaining extreme values of derivatives of the Riemann zeta function has been studied. In [28], Kalmynin obtained Ω‐results for the Riemann zeta function and its derivatives $\zeta^{\left(\ell \right)}\left(\sigma +it\right)$, when $\sigma =\sigma \left(t\right)\to 1^{-}$, as t→∞.

In [40], we established Ω‐results for $|\zeta^{\left(\ell \right)}\left(\sigma +it\right)|$ when ℓ∈N and σ∈[1/2,1) are given. These results are comparable with the best currently known lower bounds for maximum of |ζ(σ+it)|. When σ=1, we obtain lower bounds different from the case of |ζ(1+it)|. Namely, we established that $\max_{T⩽t⩽2T}\left|\zeta^{\left(\ell \right)}\left(1+it\right)\right|⩾e^{\gamma }\cdot \ell^{\ell }\cdot {\left(\ell +1\right)}^{-(\ell +1)}\cdot {\left(\log_{2}T-\log_{3}T+O\left(1\right)\right)}^{\ell +1}\;$, uniformly for all positive integers $\ell ⩽\left(\log T\right)/\left(\log_{2}T\right)$, when T is sufficiently large. On the other hand, in [41], we proved that on RH, $\;|\zeta^{\left(\ell \right)}\left(1+it\right)|\ll_{\ell }{\left(\log_{2}t\right)}^{\ell +1}\;$ for sufficiently large t, where the implied constants are effectively computable. Refining methods of [40], Z. Dong and B. Wei [17] proved that $\max_{T⩽t⩽2T}\left|\zeta^{\left(\ell \right)}\left(1+it\right)\right|⩾\left(e^{\gamma }/\left(\ell +1\right)+o\left(1\right)\right)\cdot {\left(\log_{2}T\right)}^{\ell +1}\;$, uniformly for all positive integers $\ell ⩽\left(\log T\right)/\left(\log_{2}T\right)$, as T→∞. The constant $e^{\gamma }/\left(\ell +1\right)$ improve the constant $e^{\gamma }\cdot \ell^{\ell }\cdot {\left(\ell +1\right)}^{-(\ell +1)}$ by a factor ${\left(1+1/\ell \right)}^{\ell }$. However, we still have $\lim_{\ell \to \infty }e^{\gamma }/\left(\ell +1\right)=0$. In contrast, in our new result, we have $\lim_{\ell \to \infty }Y_{\ell }=\infty$. Also, we have $Y_{\ell }>e^{\gamma }/\left(\ell +1\right),\forall \ell >0$. This is due to the following identity:

$$Y_{\ell }-\frac{e^{\gamma }}{\ell +1}=\int_{1}^{\infty }\left(u^{\ell }-\frac{1}{\ell +1}\right)\rho \left(u\right)du\;,$$

and the fact that ρ(u) is always positive.

By Theorem 3, assuming RH, we have $|\zeta^{\prime \prime }\left(1+it\right)|⩽\left(12e^{\gamma }+o\left(1\right)\right){\left(\log_{2}t\right)}^{3}$ and $|\zeta^{\left(3\right)}\left(1+it\right)|⩽\left(\frac{136}{3}e^{\gamma }+o\left(1\right)\right){\left(\log_{2}t\right)}^{4}$, which improve corresponding results in [41].

The study of extreme values of *L*‐functions is an important problem in analytic number theory. Given a negative fundamental discriminant d, one can associate a primitive Dirichlet character $\chi_{d}$ (mod |d|) by defining $\chi_{d}\left(n\right)=\left(\frac{d}{n}\right)$, using the Kronecker–Legendre symbol. The value $L(1,\chi_{d})$ is related to the class number of $Q(\sqrt{d})$ via Dirichlet's class number formula

$$\begin{array}{c} L\left(1,\chi_{d}\right)=\frac{2\pi h}{\omega \sqrt{\left|d\right|}}\;, \end{array}$$

where h is the class number of $Q(\sqrt{d})$, and ω denotes the number of roots of unity in $Q(\sqrt{d})$.

Let χ be any nonprincipal character (mod q). Assuming GRH, Littlewood [31] proved that

$$L\left(1,\chi \right)∼\prod_{p⩽\log^{2}q}{\left(1-\frac{\chi (p)}{p}\right)}^{-1},\;\text{as}\;q\to \infty \;,$$

from which one can immediately obtain that $\left|L\left(1,\chi \right)\right|⩽\left(2e^{\gamma }+o\left(1\right)\right)\log_{2}q$ by Mertens' theorem. In another direction, Chowla [10] showed that there exist arbitrarily large q and nonprincipal characters χ(mod q) such that $\left|L\left(1,\chi \right)\right|⩾\left(e^{\gamma }+o\left(1\right)\right)\log_{2}q$. As in the proof of Theorem 6, the Granville–Soundararajan conjecture implies $\left|L\left(1,\chi \right)\right|⩽\left(e^{\gamma }+o\left(1\right)\right)\log_{2}q$ for nonprincipal characters χ. We mention three best results known for L(1,χ). For further information and results about extreme values of *L*‐functions, we refer to the survey [37] and [3, 14, 19, 20, 21, 29, 36]. In [21], Granville and Soundararajan established that for sufficiently large prime q and any given A⩾10, there are at least $q^{1-1/A}$ characters χ(mod q) for which

$$\left|L(1,\chi )\right|⩾e^{\gamma }\left(\log_{2}q+\log_{3}q-\log_{4}q-\log A-C\right)\;,$$

for some absolute constant C. In [3], Aistleitner, Mahatab, Munsch, and Peyrot proved that when fix ε>0, then for all sufficiently large prime q, we have

$$\max_{\chi \neq \chi_{0}}\left|L(1,\chi )\right|⩾e^{\gamma }\left(\log_{2}q+\log_{3}q-\left(1+\log_{2}4\right)-\varepsilon \right)\;.$$

In [29], when assuming GRH, Lamzouri‐X. Li–Soundararajan obtained the following upper bound for primitive character χ modulo q:

$$\begin{array}{c} \left|L(1,\chi )\right|⩽2e^{\gamma }\left(\log_{2}q-\log2+\frac{1}{2}+\frac{1}{\log_{2}q}\right)\;,\;\forall q⩾10^{10}\;. \end{array}$$

Like $L(1,\chi_{d})$, the value of the first derivative $L^{\prime }\left(1,\chi_{d}\right)$ can be related to the class number as well, namely, via the following Chowla–Selberg formula [35, page 110]:

$$\begin{array}{c} L^{\prime }\left(1,\chi_{d}\right)=-\frac{\pi }{\sqrt{\left|d\right|}}\sum_{m=1}^{\left|d\right|}\chi_{d}\left(m\right)\log\Gamma \left(\frac{m}{\left|d\right|}\right)+\frac{2h\pi (\gamma +\log2\pi )}{\omega \sqrt{\left|d\right|}}\;. \end{array}$$

In [27, page 524], Iwaniec and Kowalski mention that when assuming GRH for $L(s,\chi_{d})$, one can obtain that $|L^{\left(\ell \right)}\left(1,\chi_{d}\right)|\ll (\log_{2}{\left|d|\right)}^{\ell +1}$. However, they do not point out what the implicit constants could be. On the other hand, we do not find literatures on large values of $|L^{\left(\ell \right)}\left(1,\chi \right)|$. Theorems 2 and 4 can be considered as generalizations of theorems of Littlewood and Chowla.

The study of character sums is another central problem in number theory. In many cases, one would like to know when the following character sum is o(x):

$$\sum_{n⩽x}\chi \left(n\right)\;,$$

where χ is a nonprincipal Dirichlet character χ(mod q).

In [33], Montgomery and Vaughan show that the above character sums can be conditionally approximated by character sums over integers with small prime factors. More precisely, they prove that if χ(mod q) is nonprincipal and GRH holds, then

$$\sum_{n⩽x}\chi \left(n\right)=\Psi \left(x,y;\chi \right)+O\left(xy^{-\frac{1}{2}}\log^{4}q\right),$$

when $\log^{4}q⩽y⩽x⩽q$. One of main results in [33] states that on GRH,

(4) $$\begin{array}{c} \left|\sum_{n⩽x}\chi \left(n\right)\right|\ll \sqrt{q}\log_{2}q\;, \end{array}$$

for any nonprincipal character χ modulo q and any x. On GRH, Granville and Soundararajan [22] find an implicit constant in (4) for primitive character χ modulo q. The upper bound (4) can be used to improve the error term in the approximation formula (16) for $L^{\left(\ell \right)}\left(1,\chi \right)$. In [18], Granville and Soundararajan refine the methods of Montgomery–Vaughan to obtain the result mentioned early in the paper, which turns out to be a key to our understanding of upper bounds of $|L^{\left(\ell \right)}\left(1,\chi \right)|$. And Theorem 5 is based on the work of Montgomery–Vaughan and Granville–Soundararajan, in particular following methods of Granville–Soundararajan.

We will use Soundararajan's resonance methods [36] to prove Theorems 1 and 2. The key ingredient is the following Proposition 1.

Proposition 1 As T→∞, uniformly for all positive numbers $\ell ⩽\left(\log_{3}T\right)/\left(\log_{4}T\right)$, we have

$$\underset{r}{\sup}\left|\sum_{mk=n⩽\sqrt{T}}\frac{r\left(m\right)\bar{r(n)}}{k}{\left(\log k\right)}^{\ell }\right|/\left(\sum_{n⩽\sqrt{T}}{\left|r\left(n\right)\right|}^{2}\right)⩾\left(Y_{\ell }+o\left(1\right)\right){\left(\log_{2}T\right)}^{\ell +1},$$

where the supremum is taken over all functions r:N→C satisfying that the denominator is not equal to zero, when the parameter T is given.

In this paper, γ denotes the Euler constant. We write $\log_{j}$ for the jth iterated logarithm, so, for example, $\;\log_{2}T\;=\log\log T$, $\log_{3}T\;=\log\log\log T$. $P^{+}\left(n\right)$ denotes the largest prime factor of n. p denotes a prime number and $p_{n}$ denotes the nth prime.

## 2 PRELIMINARY RESULTS

Recall that the function $\Psi \left(x,y\right)=\#\{n⩽x|P^{+}\left(n\right)⩽y\}$ counts the number of integers n not exceed x with prime factors at most y. The Dickman function ρ(u) is a continuous function defined by the initial condition ρ(u)=1 for 0⩽u⩽1 and satisfies the following differential equation:

(5) $$\begin{array}{c} u\rho^{\prime }\left(u\right)+\rho \left(u-1\right)=0\;,\;\;u>1\;. \end{array}$$

From the definition, the Dickman function ρ(u) is a positive decreasing function. In 1930, Dickman [15] proved that for fixed u>0, $\lim_{x\to \infty }\Psi \left(x,x^{\frac{1}{u}}\right)/x$ exists and equals to ρ(u). We will use the following strong form of this asymptotic formula and an asymptotic formula for ρ(u). In the following lemma, (6) is due to Hildebrand[25]. The upper bound of (7) is due to de Bruijn[13], whereas the lower bound of (7) is due to Hildebrand[25]. And the asymptotic formula (8) for ρ(u) was obtained by de Bruijn[12].

Lemma 1 (Thm 1.1, 1.2, Cor 2.3  [26]) Let x⩾y⩾2 be real numbers, and put $u=\frac{\log x}{\log y}$. For any fixed ε>0, the asymptotic formula

(6) $$\begin{array}{c} \Psi \left(x,y\right)=x\rho \left(u\right)\left(1+O\left(\frac{\log(u+1)}{\log y}\right)\right) \end{array}$$

holds uniformly in the range $1⩽u⩽\exp({\left(\log y\right)}^{\frac{3}{5}-\varepsilon })$.  The weaker relation

(7) $$\begin{array}{c} \log\frac{\Psi (x,y)}{x}=\left(1+O\left(\exp(-{\left(\log u\right)}^{\frac{3}{5}-\varepsilon })\right)\right)\log\rho \left(u\right) \end{array}$$

holds uniformly in the range $1⩽u⩽y^{1-\varepsilon }$. And as u→∞,

(8) $$\begin{array}{c} \log\rho \left(u\right)=-u\left(\log u+\log_{2}\left(u+2\right)-1+O\left(\frac{\log_{2}\left(u+2\right)}{\log(u+2)}\right)\right)\;. \end{array}$$

The following lemma is on the Laplace transform of the Dickman function, which is useful for us to compute $Y_{\ell }$, as mentioned in Remark 1.

Lemma 2 (Lemma 2.6 [26],  Thm 7.10 [34]) For any real or complex number s, we have

$$\begin{array}{c} \int_{0}^{\infty }\rho \left(u\right)e^{-us}du=\exp\left(\gamma +\int_{0}^{s}\frac{e^{-z}-1}{z}dz\right)\;. \end{array}$$

We have the following conditional approximation formula for logζ(σ+it), which is adapted from [21, Lemma 1].

Lemma 3 (Granville–Soundararajan) Assume RH. Let y⩾2 and t⩾y+3. For $\frac{1}{2}<\sigma ⩽1$, we have

$$\log\zeta \left(\sigma +it\right)=\sum_{n=2}^{\left[y\right]}\frac{\Lambda (n)}{n^{\sigma +it}\log n}+O\left(\frac{\log t}{{\left(\sigma_{1}-\frac{1}{2}\right)}^{2}}y^{\sigma_{1}-\sigma }\right),$$

where we put $\sigma_{1}=\min\left(\frac{1}{2}+\frac{1}{\log y},\frac{\sigma }{2}+\frac{1}{4}\right)$.

We have the following unconditional approximation formula for $\zeta^{\left(\ell \right)}\left(\sigma +it\right)$. The constant 6.28 can be replaced by any positive number smaller than 2π (see [23, Lemma 2]).

Lemma 4 (Lemma 1 [40]) Let $\sigma_{0}\in \left(0,1\right)$ be fixed. If T is sufficiently large, then uniformly for ε>0, t∈[T,6.28T], $\sigma \in [\sigma_{0}+\varepsilon ,\;\infty )$ and all positive integers ℓ, we have

(9) $${\left(-1\right)}^{\ell }\zeta^{\left(\ell \right)}\left(\sigma +it\right)=\sum_{n⩽T}\frac{{\left(\log n\right)}^{\ell }}{n^{\sigma +it}}+O\left(\frac{\ell !}{\varepsilon^{\ell }}\cdot T^{-\sigma +\varepsilon }\right)\;,$$

where the implied constant in big O(·) only depends on $\sigma_{0}$.

## 3 PROOF OF PROPOSITION 1

Let T be large. Let w=π(y). Define y, b, and P(y,b) as follows:

$$y=\frac{\log T}{3{\left(\log_{2}T\right)}^{3}}\;,\;b=\left[{\left(\log_{2}T\right)}^{3}\right]\;,\;P\left(y,b\right)=\prod_{p⩽y}p^{b-1}=\prod_{i=1}^{w}p_{i}^{b-1}\;\;.$$

Note that $P\left(y,b\right)⩽\sqrt{T}$. Let M be the set of divisors of P(y,b) and define the function r:N→{0,1} to be the characteristic function of M, then

(10) $$\begin{array}{c} |\sum_{mk=n⩽\sqrt{T}}\frac{r\left(m\right)\bar{r(n)}}{k}{\left(\log k\right)}^{\ell }|/\left(\sum_{n⩽\sqrt{T}}{\left|r\left(n\right)\right|}^{2}\right)=\frac{1}{\left|M\right|}\sum_{\begin{array}{c} n\in M\\ k|n \end{array}}\frac{{\left(\log k\right)}^{\ell }}{k}=\sum_{\begin{array}{c} k\in M \end{array}}\frac{{\left(\log k\right)}^{\ell }}{k}\left(\frac{1}{\left|M\right|}\sum_{\begin{array}{c} n\in M\\ k|n \end{array}}1\right). \end{array}$$

Define $K:\;=\{k\in N|\;k⩽\exp\left(\log_{2}T\cdot \log_{3}T\right)\;,\;\;P^{+}\left(k\right)⩽y\}$ and its two subsets $K_{1}$ and $K_{2}$ to be

$$\begin{array}{cc} & K_{1}:\;=\{k\in K|\;\sum_{i=1}^{w}\alpha_{i}⩽\frac{{\left(\log_{2}T\right)}^{3}}{\log_{3}T},\;\;\;\;\text{where}\;\;k\;\;\text{has the prime factorization as}\;\;\;k=p_{1}^{\alpha_{1}}p_{2}^{\alpha_{2}}\cdots p_{w}^{\alpha_{w}}\;\},\\ & K_{2}:\;=\{k\in K|\;\sum_{i=1}^{w}\alpha_{i}>\frac{{\left(\log_{2}T\right)}^{3}}{\log_{3}T},\;\;\;\;\text{where}\;\;k\;\;\text{has the prime factorization as}\;\;\;k=p_{1}^{\alpha_{1}}p_{2}^{\alpha_{2}}\cdots p_{w}^{\alpha_{w}}\;\}. \end{array}$$

Clearly, $K_{1}$ is a subset of M. Let k be any given element of $K_{1}$, then the inner sum in (10) tends to 1, as T→∞. More precisely, we have

(11) $$\begin{array}{c} \frac{1}{\left|M\right|}\sum_{\begin{array}{c} n\in M\\ k|n \end{array}}1⩾1-\frac{2}{\log_{3}T}\;,\;\;\forall k\in K_{1}. \end{array}$$

To see this, assume that $k=p_{1}^{\alpha_{1}}p_{2}^{\alpha_{2}}\cdots p_{w}^{\alpha_{w}}$. Then

$$\frac{1}{\left|M\right|}\sum_{\begin{array}{c} n\in M\\ k|n \end{array}}1=\frac{1}{b^{w}}\sum_{\begin{array}{c} n\in M\\ k|n \end{array}}1=\frac{1}{b^{w}}\prod_{i=1}^{w}\left(b-\alpha_{i}\right)=\prod_{i=1}^{w}e^{\log\left(1-\frac{\alpha_{i}}{b}\right)}\;,$$

and (11) follows from the condition $\sum_{i=1}^{w}\alpha_{i}⩽\frac{{\left(\log_{2}T\right)}^{3}}{\log_{3}T}\;$.

Now consider upper bounds for the sum of reciprocals of elements of $K_{2}$. By Rankin's trick and dropping conditions for $\alpha_{i}$, we have

$$\begin{array}{c} \sum_{k\in K_{2}}\frac{1}{k}⩽\sum_{\alpha_{1}=0}^{\infty }\sum_{\alpha_{2}=0}^{\infty }\cdots \sum_{\alpha_{w}=0}^{\infty }\frac{1}{p_{1}^{\alpha_{1}}p_{2}^{\alpha_{2}}\cdots p_{w}^{\alpha_{w}}}\left(\sum_{i=1}^{w}\alpha_{i}\right){\left(\frac{{\left(\log_{2}T\right)}^{3}}{\log_{3}T}\right)}^{-1}\;. \end{array}$$

Next, we use the inequality $\sum_{i=1}^{w}\alpha_{i}⩽\prod_{i=1}^{w}\left(1+\alpha_{i}\right)$ and obtain

$$\begin{array}{cc} \sum_{k\in K_{2}}\frac{1}{k} & ⩽\sum_{\alpha_{1}=0}^{\infty }\frac{\alpha_{1}+1}{p_{1}^{\alpha_{1}}}\sum_{\alpha_{2}=0}^{\infty }\frac{\alpha_{2}+1}{p_{2}^{\alpha_{2}}}\cdots \sum_{\alpha_{w}=0}^{\infty }\frac{\alpha_{w}+1}{p_{w}^{\alpha_{w}}}{\left(\frac{{\left(\log_{2}T\right)}^{3}}{\log_{3}T}\right)}^{-1}\\ & ⩽\frac{\log_{3}T}{{\left(\log_{2}T\right)}^{3}}\prod_{i=1}^{w}{\left(\frac{1}{1-\frac{1}{p_{i}}}\right)}^{2}\\ & \ll \frac{\log_{3}T}{\log_{2}T}\;, \end{array}$$

where in the last inequality, we use the Mertens' theorem. By the definition of $K_{2}$, when $k\in K_{2}$, we have $\log k⩽\left(\log_{2}T\right)\cdot \left(\log_{3}T\right)$. Thus, we find that

(12) $$\begin{array}{c} \sum_{k\in K_{2}}\frac{{\left(\log k\right)}^{\ell }}{k}\ll {\left(\log_{2}T\right)}^{\ell -1}\cdot {\left(\log_{3}T\right)}^{\ell +1}\ll {\left(\log_{2}T\right)}^{\ell }\cdot \left(\log_{3}T\right),\;\forall \ell ⩽\left(\log_{3}T\right)/\left(\log_{4}T\right)\;. \end{array}$$

In order to compute a lower bound for the outer sum in (10), we first compute the sum over the set K, then by (12), we restrict the sum to over its subset $K_{1}$, which is also a subset of M. Let $R=\exp(\log_{2}T\cdot \log_{3}T)\;$. And we keep in mind that $\ell ⩽\left(\log_{3}T\right)/\left(\log_{4}T\right)$ in the following computations.

We split the sum into two parts as follows:

$$\begin{array}{c} \sum_{k\in K}\frac{{\left(\log k\right)}^{\ell }}{k}=\sum_{k⩽y}\frac{{\left(\log k\right)}^{\ell }}{k}+\sum_{\begin{array}{c} y<k⩽R\\ P^{+}\left(k\right)⩽y \end{array}}\frac{{\left(\log k\right)}^{\ell }}{k}=S_{1}+S_{2}\;. \end{array}$$

The first sum is

$$\begin{array}{c} S_{1}=\sum_{k⩽y}\frac{{\left(\log k\right)}^{\ell }}{k}=\left(\frac{1}{\ell +1}+o\left(1\right)\right){\left(\log_{2}T\right)}^{\ell +1}\;. \end{array}$$

By partial summation, the second sum is

(13) $$\begin{array}{c} S_{2}=\frac{{\left(\log R\right)}^{\ell }}{R}\Psi \left(R,y\right)-\frac{{\left(\log y\right)}^{\ell }}{y}\Psi \left(y,y\right)-\int_{y}^{R}\frac{d}{dx}\left(\frac{{\left(\log x\right)}^{\ell }}{x}\right)\Psi \left(x,y\right)dx\;. \end{array}$$

By (6), we have

(14) $$\begin{array}{c} \Psi \left(x,y\right)=x\;\rho \left(\frac{\log x}{\log y}\right)\left(1+O\left(\frac{\log_{4}T}{\log_{2}T}\right)\right)\;,\;\text{uniformly for}\;\;y⩽x⩽R. \end{array}$$

Applying (14) into (13), and using (5) and (8), we obtain

$$\begin{array}{c} S_{2}=\left(\int_{1}^{\infty }u^{\ell }\rho \left(u\right)du+o\left(1\right)\right){\left(\log_{2}T\right)}^{\ell +1}\;. \end{array}$$

We immediately get

$$\begin{array}{c} \sum_{k\in K}\frac{{\left(\log k\right)}^{\ell }}{k}=S_{1}+S_{2}=\left(\int_{0}^{\infty }u^{\ell }\rho \left(u\right)du+o\left(1\right)\right){\left(\log_{2}T\right)}^{\ell +1}\;. \end{array}$$

Together with (12), we have

(15) $$\begin{array}{c} \sum_{k\in K_{1}}\frac{{\left(\log k\right)}^{\ell }}{k}=\left(\int_{0}^{\infty }u^{\ell }\rho \left(u\right)du+o\left(1\right)\right){\left(\log_{2}T\right)}^{\ell +1}\;. \end{array}$$

Since $K_{1}$ is a subset of M, we find that

$$\begin{array}{c} \sum_{\begin{array}{c} k\in M \end{array}}\frac{{\left(\log k\right)}^{\ell }}{k}\left(\frac{1}{\left|M\right|}\sum_{\begin{array}{c} n\in M\\ k|n \end{array}}1\right)⩾\sum_{\begin{array}{c} k\in K_{1} \end{array}}\frac{{\left(\log k\right)}^{\ell }}{k}\left(\frac{1}{\left|M\right|}\sum_{\begin{array}{c} n\in M\\ k|n \end{array}}1\right)\;. \end{array}$$

By (10), (11), and (15), we are done.□

## 4 PROOF OF THEOREM 1

By [40, page 496], we have

$$\begin{array}{cc} \max_{T⩽t⩽2T}\left|\zeta^{\left(\ell \right)}\left(1+it\right)\right| & ⩾\left(1+O(T^{-1})\right)\left|\sum_{mk=n⩽\sqrt{T}}\frac{r\left(m\right)\bar{r(n)}}{k}{\left(\log k\right)}^{\ell }\right|/\left(\sum_{n⩽\sqrt{T}}{\left|r\left(n\right)\right|}^{2}\right)\\ & +\;O\left(T^{-\frac{3}{2}}\;{\left(\log T\right)}^{\ell +1}\right)+O\left({\left(\log_{2}T\right)}^{\ell }\right)\;. \end{array}$$

By Proposition 1, we finish the proof of Theorem 1.□

## 5 PROOF OF THEOREM 2

First, note that we have the following approximation formula by partial summation and Pólya–Vinogradov inequality ([34, Thm 9.18])

(16) $$\begin{array}{c} L^{\left(\ell \right)}\left(1,\chi \right)=\sum_{k⩽N}\frac{\chi \left(k\right){\left(-\log k\right)}^{\ell }}{k}+O\left(\frac{\sqrt{q}\log q{\left(\log N\right)}^{\ell }}{N}\right)\;,\;\text{when}\;\chi \neq \chi_{0}\;,\;\ell ⩽\log N\;. \end{array}$$

In order to use Soundararajan's resonance method [36] to produce extreme values, we define $V_{2}\left(q\right)$ and $V_{1}\left(q\right)$ as follows (also see [14, page 129]):

$$\begin{array}{c} V_{2}\left(q\right):\;=\sum_{\chi \neq \chi_{0}}{\left(-1\right)}^{\ell }L^{\left(\ell \right)}\left(1,\chi ;N\right){\left|R_{\chi }\right|}^{2}\;,\;V_{1}\left(q\right):\;=\sum_{\chi \neq \chi_{0}}{\left|R_{\chi }\right|}^{2}\;, \end{array}$$

where $L^{\left(\ell \right)}\left(1,\chi ;N\right)$ and the resonator $R_{\chi }$ are defined by

$$\begin{array}{c} L^{\left(\ell \right)}\left(1,\chi ;N\right):\;=\sum_{k⩽N}\frac{\chi \left(k\right){\left(-\log k\right)}^{\ell }}{k}\;,\;R_{\chi }:\;=\sum_{a⩽A}\chi \left(a\right)r\left(a\right)\;. \end{array}$$

We chose $T=q^{\frac{1}{2}}$, $N=q^{\frac{3}{4}}$, $A=q^{\frac{1}{4}}$ and we let the function r(n) to be defined as in the proof of Proposition 1. By orthogonality of characters, we have

(17) $$\begin{array}{c} V_{1}\left(q\right)⩽\sum_{\chi }{\left|R_{\chi }\right|}^{2}⩽\phi \left(q\right)\sum_{a⩽A}r\left(a\right)\;. \end{array}$$

By Cauchy's inequality, we have

$${\left|R_{\chi_{0}}\right|}^{2}⩽A\sum_{a⩽A}r\left(a\right)\;.$$

Thus, we can bound $|L^{\left(\ell \right)}\left(1,\chi_{0};N\right)\left|\cdot \right|R_{\chi_{0}}{|}^{2}$ by

$$\begin{array}{c} ⩽{\left(\log q\right)}^{\ell +1}A\sum_{a⩽A}r\left(a\right). \end{array}$$

Above upper bound together with the orthogonality of characters gives that

(18) $$\begin{array}{c} V_{2}\left(q\right)=\phi \left(q\right)\sum_{mk=n⩽A}\frac{{\left(\log k\right)}^{\ell }r\left(m\right)r\left(n\right)}{k}+O\left({\left(\log q\right)}^{\ell +1}\right)\cdot A\sum_{a⩽A}r\left(a\right)\;. \end{array}$$

Combining (18) with (17), we have

(19) $$\begin{array}{c} \max_{\chi \neq \chi_{0}}\left|L^{\left(\ell \right)}\left(1,\chi ;N\right)\right|⩾\left|\frac{V_{2}\left(q\right)}{V_{1}\left(q\right)}\right|=\left(\sum_{mk=n⩽A}\frac{{\left(\log k\right)}^{\ell }r\left(m\right)r\left(n\right)}{k}\right)/\left(\sum_{a⩽A}r\left(a\right)\right)+O\left({\left(\log q\right)}^{\ell +1}\right)\cdot q^{-\frac{3}{4}}\;. \end{array}$$

By (19), (16), and Proposition 1, we obtain (1).□

## 6 PROOF OF THEOREM 3

Let $x_{1}=\exp\left({\left(\log_{2}T\right)}^{2}\right)$, $x_{2}=T$, and $y_{j}=\log^{2}T\log^{2}x_{j}\;{\left(\log_{2}T\right)}^{12}$ for j=1,2. Note that we have $\log y_{1}∼2\log_{2}T$, as T→∞. By taking σ=1 and $\varepsilon ={\left(\log_{2}T\right)}^{-1}$ in (9), we have

$${\left(-1\right)}^{\ell }\zeta^{\left(\ell \right)}\left(1+it\right)=\sum_{k⩽T}\frac{{\left(\log k\right)}^{\ell }}{k^{1+it}}+O\left({\left(\log_{2}T\right)}^{\ell }\right),\;\forall t\in \left[2T,\;5T\right]\;.$$

We split the sum in the above approximation formula into two parts as follows:

$$\begin{array}{c} \sum_{k⩽x_{2}}\frac{{\left(\log k\right)}^{\ell }}{k^{1+it}}=\sum_{k⩽y_{1}}\frac{{\left(\log k\right)}^{\ell }}{k^{1+it}}+\sum_{y_{1}<k⩽x_{2}}\frac{{\left(\log k\right)}^{\ell }}{k^{1+it}}. \end{array}$$

For the first sum, we have

$$\begin{array}{c} \left|\sum_{k⩽y_{1}}\frac{{\left(\log k\right)}^{\ell }}{k^{1+it}}\right|⩽\sum_{k⩽y_{1}}\frac{{\left(\log k\right)}^{\ell }}{k}=\left(\frac{1}{\ell +1}+o\left(1\right)\right){\left(\log y_{1}\right)}^{\ell +1}=\left(\frac{1}{\ell +1}+o\left(1\right)\right){\left(2\log_{2}T\right)}^{\ell +1}\;. \end{array}$$

For the second sum, by partial summation, we have

$$\begin{array}{c} \sum_{y_{1}<k⩽x_{2}}\frac{{\left(\log k\right)}^{\ell }}{k^{1+it}}=\frac{{\left(\log x_{2}\right)}^{\ell }}{x_{2}}\left(\sum_{k⩽x_{2}}\frac{1}{k^{it}}\right)-\frac{{\left(\log y_{1}\right)}^{\ell }}{y_{1}}\left(\sum_{k⩽y_{1}}\frac{1}{k^{it}}\right)+\int_{y_{1}}^{x_{2}}\left(\sum_{n⩽x}\frac{1}{n^{it}}\right)\frac{d}{dx}\left(\frac{-{\left(\log x\right)}^{\ell }}{x}\right)dx\;. \end{array}$$

By (3) of Theorem 5 and Lemma 1, we have

$$\begin{array}{c} \left|\frac{{\left(\log x_{2}\right)}^{\ell }}{x_{2}}\left(\sum_{k⩽x_{2}}\frac{1}{k^{it}}\right)\right|\ll \frac{{\left(\log T\right)}^{\ell }}{T}\Psi \left(T,\log^{2}T{\left(\log_{2}T\right)}^{20}\right)\ll {\left(\log T\right)}^{\ell }\;T^{-\frac{1}{2}+o\left(1\right)}\ll o\left(1\right)\cdot {\left(\log_{2}T\right)}^{\ell +1}\;. \end{array}$$

Clearly, we have

$$\begin{array}{c} \left|\frac{{\left(\log y_{1}\right)}^{\ell }}{y_{1}}\left(\sum_{k⩽y_{1}}\frac{1}{k^{it}}\right)\right|⩽{\left(\log y_{1}\right)}^{\ell }⩽o\left(1\right)\cdot {\left(\log_{2}T\right)}^{\ell +1}\;. \end{array}$$

By (2) of Theorem 5, (5), and Lemma 1, we have

$$\begin{array}{cc} \left|\int_{y_{1}}^{x_{1}}\left(\sum_{n⩽x}\frac{1}{n^{it}}\right)\frac{d}{dx}\left(\frac{-{\left(\log x\right)}^{\ell }}{x}\right)dx\right| & ⩽\int_{y_{1}}^{x_{1}}\left(1+o(1)\right)\Psi \left(x,y_{1}\right)\frac{d}{dx}\left(\frac{-{\left(\log x\right)}^{\ell }}{x}\right)dx\\ & ⩽\left(\int_{1}^{\infty }u^{\ell }\rho \left(u\right)du+o\left(1\right)\right){\left(2\log_{2}T\right)}^{\ell +1}\;. \end{array}$$

Again, by (2), (5), and Lemma 1, we have

$$\begin{array}{cc} \left|\int_{x_{1}}^{x_{2}}\left(\sum_{n⩽x}\frac{1}{n^{it}}\right)\frac{d}{dx}\left(\frac{-{\left(\log x\right)}^{\ell }}{x}\right)dx\right| & ⩽\int_{x_{1}}^{x_{2}}\left(1+o(1)\right)\Psi \left(x,y_{2}\right)\frac{d}{dx}\left(\frac{-{\left(\log x\right)}^{\ell }}{x}\right)dx\\ & ⩽o\left(1\right)\cdot {\left(\log_{2}T\right)}^{\ell +1}\;. \end{array}$$

As a result, we obtain

$$\begin{array}{cc} \left|\zeta^{\left(\ell \right)}\left(1+it\right)\right| & ⩽\left(\frac{1}{\ell +1}+o\left(1\right)\right){\left(2\log_{2}T\right)}^{\ell +1}+\left(\int_{1}^{\infty }u^{\ell }\rho \left(u\right)du+o\left(1\right)\right){\left(2\log_{2}T\right)}^{\ell +1}\\ & ⩽\left(2^{\ell +1}\;Y_{\ell }+o\left(1\right)\right){\left(\log_{2}T\right)}^{\ell +1}\;. \end{array}$$

Since t∈[2T,5T], we are done.□

## 7 PROOF OF THEOREM 5

The proof is almost the same as the proof of the Granville–Soundararajan theorem [18, page 389–391]. Only a few modifications are needed.

Define

$$\zeta \left(s;y\right)=\zeta \left(s\right)\prod_{p⩽y}\left(1-\frac{1}{p^{s}}\right)\;,$$

so that ζ(s;y) is a meromorphic function on the whole plane, which is holomorphic everywhere except for a simple pole at s=1. When |Im(s)|⩽T and $T+y+3⩽t⩽T^{1000}$, we have $y+3⩽\text{Im}\left(s+it\right)⩽T+T^{1000}$. Note that

$$\begin{array}{cc} \log\zeta (s+it;y) & =\log\zeta \left(s+it\right)-\sum_{p⩽y}\frac{1}{p^{s+it}}+O\left(\sum_{p⩽y}\frac{1}{p^{2\text{Re}(s)}}\right), \end{array}$$

and so, if 1> Re$\left(s\right)⩾\frac{1}{2}+\frac{1}{\log y}$, $T+y+3⩽t⩽T^{1000}$,  y⩾2 , and |Im(s)|⩽T, we get by Lemma 3

$$\left|\log\zeta (s+it;y)\right|⩽C\log T\log^{2}y,$$

where C>0 is some constant. Now suppose that $x\in N+\frac{1}{2}$. Let $u=\frac{\log x}{\log y}$ and put $c=1+\frac{1}{\log x}$. By Perron's formula,

$$\begin{array}{cc} \sum_{n⩽x}\frac{1}{n^{it}}-\Psi \left(x,y;t\right) & =\frac{1}{2\pi i}\int_{c-i\infty }^{c+i\infty }\prod_{p⩽y}{\left(1-\frac{1}{p^{s+it}}\right)}^{-1}\left(\exp\left(\log\zeta (s+it;y)\right)-1\right)\frac{x^{s}}{s}ds\\ & =\sum_{j=1}^{\left[u\right]}\frac{1}{j!}\sum_{\begin{array}{c} n⩽x/y^{j}\\ P^{+}\left(n\right)⩽y \end{array}}\frac{1}{n^{it}}\frac{1}{2\pi i}\int_{c-i\infty }^{c+i\infty }{\left(\log\zeta (s+it;y)\right)}^{j}{\left(\frac{x}{n}\right)}^{s}\frac{ds}{s}. \end{array}$$

Note that $\log\zeta \left(s;y\right)=\sum_{n=2}^{\infty }\Lambda_{y}\left(n\right){\left(\log n\right)}^{-1}n^{-s}$, where the generalized von Mangoldt function $\Lambda_{y}\left(\cdot \right)$ is defined as $\Lambda_{y}\left(n\right)=\log p$ if $n=p^{k}$ and p>y, otherwise $\Lambda_{y}\left(n\right)=0$.

So, we have ${\left(\log\zeta \left(s+it;y\right)\right)}^{j}/j!=\sum_{m=1}^{\infty }a_{j}\left(m,y\right)m^{-s-it}$, where $|a_{j}\left(m,y\right)|⩽1$ for all m, j, and y. All other steps are the same as the proof of the Granville–Soundararajan theorem.□

## 8 PROOF OF THEOREM 4

Let $x_{1}=\exp\left({\left(\log_{2}q\right)}^{2}\right)$, $x_{2}=q^{\frac{3}{4}}$ , and $y_{j}=\log^{2}q\log^{2}x_{j}\;{\left(\log_{2}q\right)}^{12}$ for j=1,2. We will use the approximation formula (16) for $L^{\left(\ell \right)}\left(1,\chi \right)$ and other steps are the same as the proof of Theorem 3.□

## 9 PROOF OF THEOREM 6

Let $x_{1}=\exp\left({\left(\log_{2}q\right)}^{2}\right)$, $x_{2}=q^{\frac{3}{4}}$ , and $y_{j}=\left(\log q+\log^{2}x_{j}\right){\left(\log_{2}q\right)}^{A}$ for j=1,2. We again use (16) and other steps are the same as the proof of Theorem 3. Note that now we have $\log y_{1}∼\log_{2}q$, as q→∞. Thus, in the end, we obtain $Y_{\ell }{\left(\log_{2}q\right)}^{\ell +1}$ instead of $Y_{\ell }{\left(2\log_{2}q\right)}^{\ell +1}$ in Theorem 4 .□

## 10 PROOF OF THEOREM 7

For the upper bound, let $x_{1}=\exp\left({\left(\log_{2}T\right)}^{2}\right)$, $x_{2}=T$, and $y_{j}=\left(\log T+\log^{2}x_{j}\right){\left(\log_{2}T\right)}^{A}$ for j=1,2. And other steps are the same as the proof of Theorem 3. Combining with the lower bound, we are done.□

## 11 A MIXED CONJECTURE

Combining the Granville–Soundararajan conjecture and Conjecture 1, we pose the following mixed conjecture.

Conjecture 2 There exists a constant A>0 such that for any nonprincipal character χ (mod q), and for any 1⩽x⩽min{q,T}, 2T⩽t⩽5T, we have, uniformly,

$$\sum_{n⩽x}\frac{\chi (n)}{n^{it}}=\sum_{\begin{array}{c} n⩽x\\ P^{+}\left(n\right)⩽y \end{array}}\frac{\chi (n)}{n^{it}}+o\left(\Psi \left(x,y;\chi_{0}\right)\right),\;\text{as}\;q\to \infty \;,T\to \infty \;,$$

where $y=\left(\log qT+\log^{2}x\right){\left(\log_{2}qT\right)}^{A}$.

## JOURNAL INFORMATION

The *Bulletin of the London Mathematical Society* is wholly owned and managed by the London Mathematical Society, a not‐for‐profit Charity registered with the UK Charity Commission. All surplus income from its publishing programme is used to support mathematicians and mathematics research in the form of research grants, conference grants, prizes, initiatives for early career researchers and the promotion of mathematics.
